# A Comprehensive Analysis of the Lipidomic Signatures in *Rhizopus delemar*

**DOI:** 10.3390/jof10110760

**Published:** 2024-11-01

**Authors:** Basharat Ali, Anshu Chauhan, Mohit Kumar, Praveen Kumar, Hans Carolus, Celia Lobo Romero, Rudy Vergauwen, Ashutosh Singh, Atanu Banerjee, Amresh Prakash, Shivaprakash M. Rudramurthy, Patrick Van Dijck, Ashraf S. Ibrahim, Rajendra Prasad

**Affiliations:** 1 Amity Institute of Integrative Science and Health, Amity University Gurugram, Gurugram 122413, India; 2 Amity Institute of Biotechnology, Amity University Gurugram, Gurugram 122413, India; 3 School of Life Sciences, Jawaharlal Nehru University, New Delhi 110067, India; 4 Yeast Biofuel Group, International Centre for Genetic Engineering and Biotechnology, New Delhi 110067, India; 5 Laboratory of Molecular Cell Biology, Department of Biology, KU Leuven, 3000 Leuven, Belgium; 6 Department of Biochemistry, University of Lucknow, Lucknow 226007, India; 7 Department of Medical Microbiology, Postgraduate Institute of Medical Education & Research, Chandigarh 160012, India; 8 Division of Infectious Diseases, The Lundquist Institute at Harbor-UCLA Medical Center, Torrance, CA 90502, USA; 9 David Geffen School of Medicine, UCLA, Los Angeles, CA 90095, USA

**Keywords:** mucormycosis, drug resistance, posaconazole, amphotericin B, sphingolipids, phospholipids, sterols, mass spectrometry

## Abstract

Certain species of Mucorales have been identified as causative agents of mucormycosis, a rare yet often lethal fungal infection. Notably, these fungi exhibit intrinsic resistance to common azole drugs, which target lipids. Given the pivotal role of lipids in drug resistance and their contribution to innate resistance to azoles, this study provides a comprehensive overview of key lipid classes, including sphingolipids (SLs), glycerophospholipids (GPLs), and sterols, in *Rhizopus delemar* 99-880, a well-characterized reference strain among Mucorales. Using shotgun lipidomics as well as liquid- and gas-chromatography-based mass spectrometric analyses, we identified the lipid intermediates and elucidated the biosynthetic pathways of SLs, PGLs, and sterols. The acidic SLs were not found, probably because the acidic branch of the SL biosynthesis pathway terminates at α-hydroxy phytoceramides, as evident by their high abundance. Intermediates in the neutral SL pathway incorporated higher levels of 16:0 fatty acid compared to other pathogenic fungi. A strikingly high phosphatidylethanolamine (PE)/phosphatdylcholine (PC) ratio was observed among GPLs. Ergosterol remains the major sterol, similar to other fungi, and our analysis confirms the existence of alternate ergosterol biosynthesis pathways. The total lipidomic profile of *R. delemar* 99-880 offers insights into its lipid metabolism and potential implications for studying pathogenesis and drug resistance mechanisms.

## 1. Introduction

Mucormycosis is a serious disease caused by a group of fungi called Mucorales. There are more than two dozen species of these fungi that can cause this deadly disease. The most common culprits are species from the genera *Rhizopus* and *Mucor*, with *Rhizopus arrhizus* being the most prevalent [1]. People with diabetes or neutropenia and those who have received hematopoietic or solid organ transplants are more susceptible to mucormycosis. Unfortunately, the number of diabetic patients, especially in developing countries, is increasing, which puts them at a higher risk for this disease. In fact, diabetes is a major risk factor for mucormycosis in over 70% of cases in countries like India, compared to 36–40% globally [2,3,4].

Mucormycosis is challenging because first-line therapy treatments of lipid amphotericin B formulations are often associated with severe toxicities, which in many cases, limit their use [5,6]. Importantly, Mucorales fungi exhibit higher MICs and, in many cases, resistance to the azoles (e.g., posaconazole (POSA) and isavuconazole (ISAV)) that are used as a step-down therapy for mucormycosis [7,8]. The disease is intrinsically resistant to short-tailed azoles like fluconazole (FLC) and voriconazole (VCZ), which otherwise are the most widely used antifungal drugs [9].

Studies on azole resistance mechanisms in various pathogenic fungi have revealed similarities across different species. These mechanisms often involve the overexpression of efflux pumps, mutations in the target gene *ERG11*, or the increased expression of target enzyme lanosterol 14α-demethylase (LDM) [10,11]. It is important to note that the prevalence of these resistance mechanisms can vary among different fungi. For example, in *Candida* species, the most common mechanism is the overexpression of efflux pump genes, while in *Aspergillus fumigatus*, mutations in the azole target enzyme lanosterol 14α-demethylase (LDM) are more prevalent [12]. *Cryptococcus* species have been found to exhibit the overexpression of various genes due to reversible chromosomal duplications, point mutations in *ERG11*, and increased efflux pump activity [13,14]. In contrast, the molecular basis of drug resistance in Mucorales is not well understood and remains an area that requires further exploration. Studies have shown that the genome of *Mucor circinelloides* contains eight potential PDR-type transporters, and the regulation of these genes appears to be interconnected. Both Pdr1 and Pdr2 have been found to contribute to the resistance of *M. circinelloides* against certain azole drugs, like PCZ, ICZ, and ravuconazole (RVZ). However, it is worth noting that azole resistance in general cannot be fully explained solely by the activity of these tested efflux pumps. The involvement of other PDR genes and novel mechanisms cannot be ruled out and may play a role in drug resistance [7].

Azoles represent a crucial class of medications targeting lipids, specifically lanosterol 14α-demathylase (LDM), an enzyme encoded by the *ERG11* gene. By inhibiting LDM, azole drugs impede the production of ergosterol, a vital component of fungal cell membranes, resulting in the build-up of toxic sterols [15]. While our comprehension of acquired resistance to triazole drugs in fungi like *Aspergillus* and *Candida* species is substantial, the inherent resistance of Mucorales to short-tailed triazoles such as VCZ and FLC warrants further exploration [16]. In many fungal species, acquired azole resistance often arises from specific mutations in the LDM gene, reducing their affinity for certain triazole drugs. However, the absence of the in vitro activity of FLC and VCZ against Mucorales suggests their resistance may be innate rather than acquired, possibly due to ancient amino acid substitutions. A 2017 study by Rita Caramalho et al. aligned LDM sequences from six Mucorales species, identifying a conserved Y129F substitution that is likely responsible for innate resistance to short-tailed azoles like FLC and VCZ [17]. An analysis based on the known LDM structure indicates a mechanism for this mutation involving the disruption of a water-mediated hydrogen bond [17]. Significantly, introducing this mutation into the homologous protein of *Saccharomyces cerevisiae* conferred resistance to short-tailed azoles [18]. Other point mutations in genes involved in ergosterol biosynthesis have also been observed in Mucorales, raising concerns about the implications of emerging resistance to current antifungal drugs. Further research is needed to fully understand the mechanisms of innate resistance in Mucorales and to develop effective strategies for combating this resistance.

Research has highlighted the significant role of lipids, the target of common antifungal drugs, in determining drug susceptibility and virulence in various pathogenic fungi [19]. However, this aspect remains poorly understood in Mucorales. Considering the severity of Mucorales infections as secondary complications in COVID-19 and diabetes patients [20], it becomes imperative to gain a comprehensive understanding of the lipid landscape and imbalances to facilitate the development of effective treatments. In this study, we selected the genome-sequenced *Rhizopus delemar* 99-880 strain as a reference strain to investigate the lipidomic landscape of Mucorales [21]. Utilizing high-throughput liquid chromatography–electrospray ionization mass spectrometry (LC-ESI/MSMS) and gas chromatography coupled to mass spectrometry (GCMS) approaches, we successfully conducted qualitative and quantitative analyses of a wide range of lipids, including sphingolipids (SLs), glycerophospholipids (GPLs), and sterols. This dataset presents, for the first time, a comprehensive overview of the lipidomic profile of the well-defined *R. delemar* 99-880 strain, which is anticipated to serve as a foundational platform for future investigations in this field.

## 2. Materials and Methods

### 2.1. Strains, Media, and Growth Conditions

*R. delemar* 99-880 spores were revived from archived glycerol stocks stored at −80 °C. Spores were inoculated on YPD plates and incubated for 3–4 days until mycelia were grown and fresh spores were formed. Plates were flooded with 10 mL phosphate buffered saline with 0.01% tween (PBST), and spores were collected by aspiration. Spores were counted using hemocytometer and adjusted to a density of 10^6^ spores mL^−1^ for further experimental use.

### 2.2. Drug Susceptibility

Approximately 10^3^ spores were spotted on YPD plates containing different concentrations of Myriocin (MYR) (Sigma, Kawasaki, Japan) or Aureobasidin A (AbA) (Takara, Kusatsu, Japan). Plates were incubated at 30 °C for 48 h and then imaged (Bio-rad Molecular Imager^®^, Hercules, CA, USA).

### 2.3. Sphingolipid Isolation

Spores were inoculated in 100 mL YPD and grown at 30 °C with constant shaking until mycelia were formed (~24 h). Mycelia were harvested and washed with 0.9% saline and dried. Approximately 100 mg of mycelia was used for SL isolation in three steps [22]. Briefly, mycelia were homogenized in 1 mL Mandala buffer (ethanol:dH_2_O:diethyl ether:pyridine:NH_4_OH = 15:15:5:1:0.018; *v*/*v*]) using glass beads. C17 sphingosine and C17 ceramide (Avanti Polar Lipids Inc., Alabaster, AL, USA) were added as internal standards before isolation. The lysate was heated at 60 °C for 30 min and then centrifuged. The upper layer was separated using Pasteur pipette and dried with periodic N_2_ flushing. Next Bligh and Dyer extraction was performed in which the dried lipid pellet was dissolved in methanol:chloroform (2:1) and incubated for 1 h at 37 °C. To this, chloroform–dH_2_O was added at a ratio of 1:1 for phase separation. The lower layer was separated by centrifugation, transferred to other tube, and dried. Finally, mild alkaline hydrolysis was performed by adding 0.5 mL chloroform and 0.5 methanolic KOH (0.6M) and then vortexing and incubating the mixture for 1 h at room temperature. To this, 0.325 mL of 1N HCl and 0.125 mL of dH_2_O were added and the mixture was vortexed again. The layers were then separated by centrifugation, and lower layer was pipetted out and dried in presence of N_2_ gas. Dried SL extracts were dissolved in 300 µL organic buffer (methanol with 1 mM ammonium formate and 0.2% formic acid). From this, 20 µL sample was transferred to glass insert and final volume was adjusted to 200 µL in organic buffer for LC-MS/MS analysis.

### 2.4. Phospholipid Isolation

PLs were extracted using the Folch method with slight modifications [23]. Equisplash^®^ (Avanti Polar Lipids Inc., Alabaster, AL, USA) containing an equal proportion of deuterated PLs for each class was added as internal standard before isolation. Briefly, 100 mg mycelia were homogenized in 1 mL LC-MS grade water using glass beads in FastPrep (MP Biomedicals, Santa Ana, CA, USA). To this lysate, 9 mL of chloroform–methanol (2:1) was added and incubated for 2 h with periodic vortexing. The upper layer was discarded, and 2 mL of 0.9% saline was added. Lower layer was then separated by centrifugation at 3000 rpm for 5 min and dried with N_2_ gas. Dried extracts were dissolved in 1 mL organic buffer. From this, 10 µL sample was transferred to glass insert, and final volume was adjusted to 200 µL in organic buffer for LC-MS/MS analysis.

### 2.5. Sterol Extraction

Harvested mycelia were finely crushed in liquid N_2_, and approx. 20 mg was weighed and used for sterol extraction. Sterols were extracted as described by Morio et al. [24]. Mycelia was resuspended in 300 µL saponification solution containing 12.5 g KOH dissolved in 18 mL MQ water and adjusted to 50 mL with 98% ethanol. The mixture was then heated at 80 °C for 1 h in a capped glass vial. After cooling at RT, 100 µL MQ water, 400 µL hexane, and 1 µL cholestane (5 mg/mL) as internal standard were added. The mixture was vortexed, and after phase separation, the top layer of ~350 µL (hexane) was separated and 600 µL hexane was again added to the remaining lysate. After vortexing and phase separation, 550 µL of the top layer was separated and both hexane fractions were combined and dried using vacuum centrifugation at room temperature. The dried sterol extracts were re-dissolved in a mixture of 60 µL hexane and 10 µL Silylation mixture (Sigma) for derivatization, vortexed, and incubated for 1 h at room temperature. From this, 50 µL of the debris-free extract was transferred into glass inserts for GCMS analysis.

### 2.6. Liquid Chromatography–Mass Spectrometry

SLs were analyzed by employing shotgun and multiple reaction monitoring (MRM) approaches using LC-MS (QTRAP^®^ 4500, AB SCIEX, Framingham, MA, USA). For untargeted shotgun lipidomics, different confirmatory scans were used, and the spectra obtained are given in Supplementary File S1. For the targeted MRM approach, SLs and PLs were separated on C8 and C18 columns maintained at 60 °C and 50 °C, respectively (Waters, Milford, MA, USA). Organic buffer (methanol with 1 mM ammonium formate and 0.2% formic acid) and aqueous buffer (water with 2 mM ammonium formate and 0.2% formic acid) were used as mobile phase. A combined flow rate of 300 µL per min was maintained in a gradient manner starting with 80% organic buffer, gradually increased to 88%, then 99%, decreased to 88% and finally restored to 80% before final run. Mass spectrometric parameters were standardized as follows: source temperature was set at 600 °C for SL; nebulizer and desolvation gases (GS1 and GS2, respectively) were set at 50 psi each; electrospray ionization voltage was set at 5500 V; and curtain gas was set at 45 psi. For PL analysis, the source temperature was set as 400 °C, GS1 and GS2 were set at 45 psi, electrospray ionization voltage was set at 4500 V, and curtain gas was set at 45 psi.

### 2.7. Gas Chromatography–Mass Spectrometry

Derivatized sterol samples were analyzed using Thermo Scientific GCMS system (Trace 1300 ISQ QD, Thermo Scientific, Waltham, MA, USA). Samples were injected in split mode at 250 °C with a ratio of 1:10, and helium at a flow rate of 1.4 mL per min was used as carrier gas. Starting temperature was held at 50 °C for 1 min, ramped up at a rate of 50 °C/min to 260 °C, ramped up again at 2 °C/min to 325 °C, and held for 3 min. Masses were detected within a range of 50 to 600 atomic mass units using electron impact ionization (70 eV). Transfer line and the detector were operated at 325 °C and 250 °C, respectively.

Different sterol intermediates were identified by their retention times with respect to the internal standard cholestane and fragmentation patterns using Chromeleon 7 (Thermo Scientific). The spectra obtained were matched to NIST library and those described by Muller et al. [25]. The relative abundance was calculated from the peak area of each sterol molecule normalized to the internal standard signal.

### 2.8. Protein Estimation

An aliquot of 25 µL was taken from lysate of each replicate for total protein estimation in order to normalize the quantified data. Protein estimation was performed using bicinchoninic acid (BCA) protein assay kit (G-Biosciences, St. Louis, MO, USA) as described previously [26].

### 2.9. Data Analysis

Mass spectrometric chromatograms of SL and PL molecules were analyzed using MultiQuant™ ver. 3.0.3 software (SCIEX). As mentioned earlier, separate internal standards for SLs and PLs were used to normalize the area of each lipid species. Three biological replicates were run for all analyses, and data are presented as % of total SL, PL, or sterol content. Data were plotted using GraphPad Prism 8. Structures of lipid molecules were drawn using structure drawing tools available on https://www.lipidmaps.org/resources/tools/structure (accessed on 28 July 2024). The lipid biosynthesis pathways were assembled based on the presence of (i) orthologous genes involved in lipid biosynthesis in *R. delemar* genome [21]; and (ii) presence of lipid intermediates detected by mass spectrometry analysis. The pathways were assembled based on known biosynthetic pathways in other fungi [22,27,28,29,30,31,32,33].

## 3. Results

### 3.1. R. delemar Harbours All Major SL Classes

**Figure 1:**
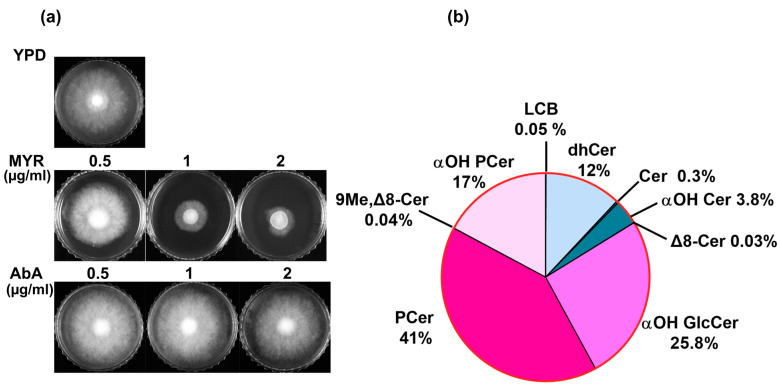
(**a**) Susceptibility of *R. delemar* to SL inhibitors MYR and AbA. Approx. 10^3^ spores were spotted on YPD plates with different concentrations of MYR and AbA. Plates were imaged after incubation at 30 °C for 48 h. (**b**) Pie chart represents proportion of different SL intermediates in *R. delemar*.

The relevance of SLs in *R. delemar* became apparent when we checked its susceptibility towards specific inhibitors. *R. delemar* cells were susceptible to myriocin, a potent inhibitor of serine palmitoyltransferase (SPT), which is the enzyme responsible for the first and rate-limiting step in SL biosynthesis (Figure 1a). By inhibiting SPT, myriocin disrupts SLs’ homeostasis in yeast cells, which can have various downstream effects on cell physiology and function [34]. On the other hand, *R. delemar* cells were resistant to aureobasidin A (AbA), a cyclic depsipeptide antibiotic that inhibits inositol phosphorylceramide (IPC) synthase encoded by the *AUR1* gene (Figure 1a). The resistance to AbA indicated a lack of an acidic SL pathway in *R. delemar* cells (as discussed below). Our susceptibility profile of *R. delemar* underscores the relevance of SLs in its various cellular processes, which prompted us to analyze SLs’ composition in detail. SLs were detected in base hydrolyzed lipid samples by the shotgun and MRM approaches, as described previously [22,27]. Multiple confirmatory scans specific for the detection of different SL intermediates were performed in positive mode ([M + H]^+^). Since it was the first high throughput analysis of this fungal pathogen, we subjected the samples to unbiased scanning over a mass range of *m*/*z* of 200 to 1000 dalton (Da), which included most of the commonly found SLs in fungi. Separate chromatograms were recorded for each type of scan, which included numerous masses. The mass signals obtained in each chromatogram corresponded to SL molecular species representing the different SL classes, which are structurally unique from each other. The different confirmatory scans and the detected SL intermediates are given in Supplementary File S1.

Our analyses of the detected masses enabled us to identify all diverse classes of SLs that differed based on headgroup, the presence of double bonds, and the number of hydroxyl groups on the sphingoid base (backbone). These included commonly found SL classes such as long-chain bases (LCBs), dihydroceramides (dhCer), ceramides (Cer), alpha-hydroxy ceramides (α-OH Cer), alpha-hydroxy glucosylceramides (α-OH GlcCer), phytoceramides (PCer), and alpha-hydroxy phytoceramides (α-OH PCer) [22,26,27]. In addition, fungal-specific lipids such as Δ8-ceramide (Δ8-Cer) and 9-methyl, Δ8-ceramide (9-Me, Δ8-Cer) were also detected (Figure 1b). Additionally, all the molecular species detected had five types of backbones. Also, the fatty acids acylated to the sphingoid base were either saturated or mono-unsaturated, hydroxylated (likely at the C2 position), or non-hydroxylated, thus generating multiple pools of SL species in combination. Selected masses from the shotgun approach were further detected via MRMs, and the samples were subjected to LC-ESI/MSMS for the separation and quantification of each lipid species. The peak identification and quantification of each species were performed on the basis of the retention time (RT) with respect to available natural SL standards. The complete list of identified SL species and their relative abundance are given in Supplementary File S2.

#### 3.1.1. Long-Chain Base (LCBs) Content Is Very Low with DHS-1-P as the Major Base

**Figure 2:**
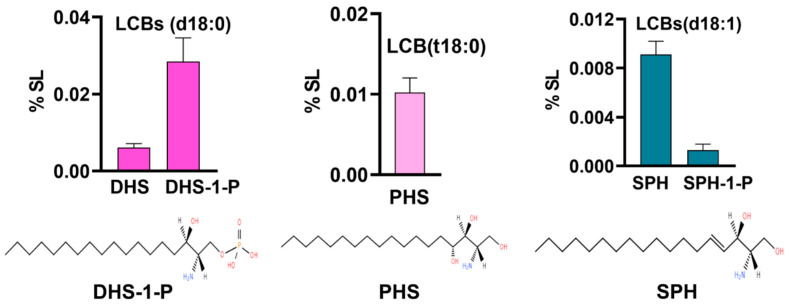
LCBs detected in *R. delemar*: DHS, DHS-1-P(d18:0), PHS(t18:0) and SPH, SPH-1-P(d18:1). Bar graphs represent relative proportion of these bases out of total SL content. Structures represent top three major bases, each with different backbone.

Our analysis could detect all major LCBs including dihydrosphingosine (DHS), phytosphingosine (PHS), sphingosine (SPH), dihydrosphingosine-1-phosphate (DHS-1-P), and sphingosine-1-phosphate (SPH-1-P). Notably, glucosyl-sphingosine (Glu-SPH) and phytosphingosine-1-phosphate (PHS-1-P) could not be detected in *R. delemar* cells (Figure 2). In combination, the total LCB content was very low (0.05%) with DHS-1-P emerging as a major base (0.02%) followed by PHS (0.01%), both sharing approximately two-thirds of the LCB content between them. Other bases such as DHS, SPH, and SPH-1-P were present in trace amounts ranging between 0.001% and 0.009%.

#### 3.1.2. dhCer Synthesis Leads the Initiation of Neutral Pathway and 16:0 Is the Major Species

**Figure 3:**
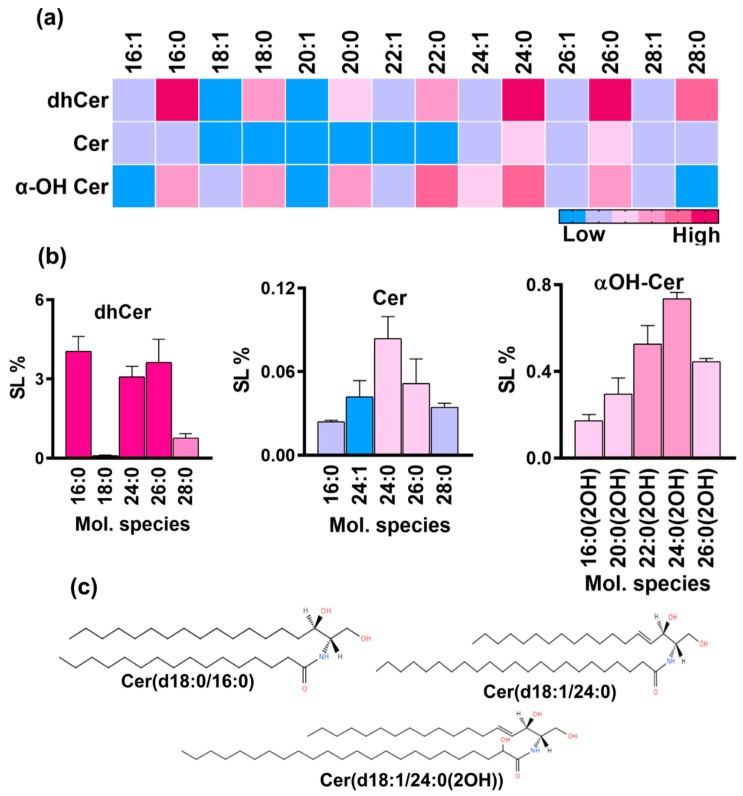
SL classes and molecular species of the neutral branch of SL biosynthesis pathway in *R. delemar*. (**a**) Heatmap represents the relative representation of different molecular species of dhCer, Cer, and αOH-Cer. (**b**) Top five species of each class were quantified and are represented by bar graphs. (**c**) Structures represent the major molecular species of each class.

dhCer structures are synthesized by the acylation of the sphingoid base, DHS, through the amine group present at the C2 position of the base. These intermediates serve as starting precursors of the neutral branch of the fungal SL biosynthesis pathway. We could detect multiple species of dhCer. For the analysis, selected masses corresponding to molecular species with 16 to 28 carbon long-chain fatty acids were quantified (Figure 3a). The total dhCer content was ~12% with 16:0 dhCer as the predominant species (Figure 3b). The species with longer chain lengths of 24:0, 26:0, and 28:0 shared 3%, 3%, and 0.7% of the SL content, respectively. Other species with saturated fatty acids such as 18:0, 20:0, 22:0 were present in very low amounts, each having contributions ≤ 0.1%. Species with mono-unsaturated fatty acids were present in trace amounts.

#### 3.1.3. Cer and α-OH Cer Species Were Also Detected and 24:0 Is the Major Species in Both Classes

The desaturation of dhCer (d18:0 backbone) leads to the formation of Cer, which is a central hub acting as the precursor to many simple-to-complex SLs in eukaryotic cells. The total Cer content was ~0.3% mainly contributed by species with 16C to 28C long-chain fatty acids, out of which 24:0 was the major species (0.08%) followed by 26:0, 24:1, 28:0, 26:1, 16:0, and 16:1 (ranging between 0.02 and 0.05%) (Figure 3b). The other Cer species were found in trace amounts. Notably, species containing mono-unsaturated fatty acids were less abundant than the saturated ones with the same carbon number chain length. SLs with the same backbone as that of Cer (d18:1) but with hydroxy fatty acids most likely at the C2 position, known as αOH-Cer, were also present. The total αOH-Cer was ~3.9% of the total SL content with a wide range of species ranging from 14C to 30C long-chain fatty acids. The major species were hydroxylated 24:0, 22:0, and 20:0, contributing, 0.7%, 0.5%, and 0.4%, respectively. The other prominent species were 20:0, 16:0, 18:0, and 14:0, ranging from 0.1% to 0.29%. Other species and species containing mono-unsaturated fatty acids were present in very low amounts (<0.1%).

#### 3.1.4. *R. delemar* Harbors Typical Fungal-Specific Lipids

**Figure 4:**
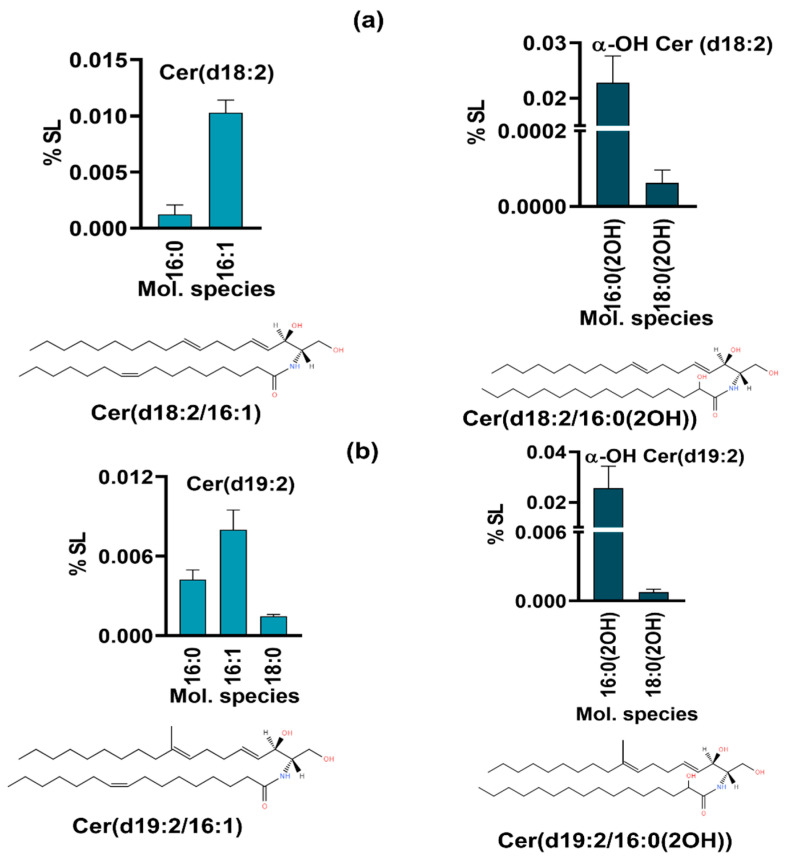
Fungal-specific SL intermediates (**a**) with d18:2(Δ8-Cer); and (**b**) with d19:2 backbone (9Me,Δ8-Cer). Bars depict the top molecular species for each backbone type. The structure of one representative species is depicted at the bottom of (**a**,**b**) panels.

The fungal-specific SLs, di-unsaturated sphingoid base, d18:2, or Δ8-Cer synthesized by the desaturation of Cer at the C8 position were detected (Figure 4) along with the downstream product, 9-Me, Δ8-Cer having a d19:2 backbone [30]. In d18:2, two Cer species with non-hydroxy fatty acids, 16:0 and 16:1, and two species of αOH-Cer, 16:0(2OH) and 18:0(2OH), were quantifiable. The former represented amounts of 0.001% to 0.01%, while the latter represented amounts of 0.02% and 0.0001%, thus again showing that 16C-containing species predominate, as in dhCer. In 9-methyl, Δ8-Cer, five species were detected, out of which three were non-hydroxy fatty acid species (d19:2 Cer) and two were α-OH Cer species (d19:2 α-OH Cer). The d19:2 Cer species include 16:0, 16:1, and 18:0, representing 0.004%, 0.01%, and 0.001%, respectively. The two α-OH Cer species, 16:0(2OH) and 18:0(OH), were 0.02% and 0.0007%, respectively. The species with d18:2 and d19:2 backbones are the precursors to neutral complex SLs such as GlcCer.

#### 3.1.5. Glucosylceramides Represent the Second Most Abundant SL Class

**Figure 5:**
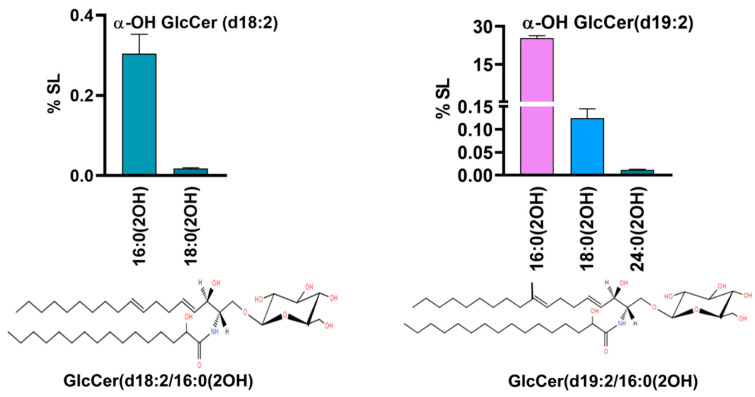
Five major species of αOH-GlcCer with d18:2 and d19:2 backbones acylated to fatty acids of varying chain lengths (*x*-axis). Structures of two major α-OH GlcCer species are drawn. GlcCer(d18:2/16:0(OH)) on the left has a backbone of 18 carbons with double bonds at C4 and C8 position, glucose at C1 position and hydroxyl group at C2 position of the 16C fatty acid chain. Similarly, on the right side, GlcCer(d19:2/16:0(OH)) is the major species with methyl group at C9 position of the sphingoid base.

As part of the neutral SL biosynthesis pathway, SL intermediates with d18:2 and d19:2 backbones are glycosylated at the C1 position to yield fungal complex SLs, such as hexosylceramides, with glucose as most common sugar in the headgroup. Our analyses detected α-OH GlcCer as a major complex SL representing ~26% of the total SL content, thus making it the second most abundant SL class in *R. delemar* after PCer. A total of five molecular species were detected that showed significant abundance (Figure 5). Two GlcCer species from the d18:2 pool had 16(2OH) and 18:0(2OH) as fatty acids, contributing 0.3% and 0.01%, respectively. In contrast, three GlcCer species from the d19:2 pool had a cumulatively higher abundance than the d18:2 pool, with 16:0(2OH), 18:0(2OH), and 24:0(2OH) as the major species, sharing ~25%, 0.1%, and 0.01% of the total SL content among them. In fact, the GlcCer d19:2/16:0(2OH) species was the largest among all the species detected in terms of quantity. GlcCer synthesis represents the terminal step in the neutral branch of the SL biosynthesis pathway [35].

#### 3.1.6. PCer Is the Most Abundant SL Class While Its Terminal Acidic Intermediates Are Absent in *R. delemar* Cells

**Figure 6:**
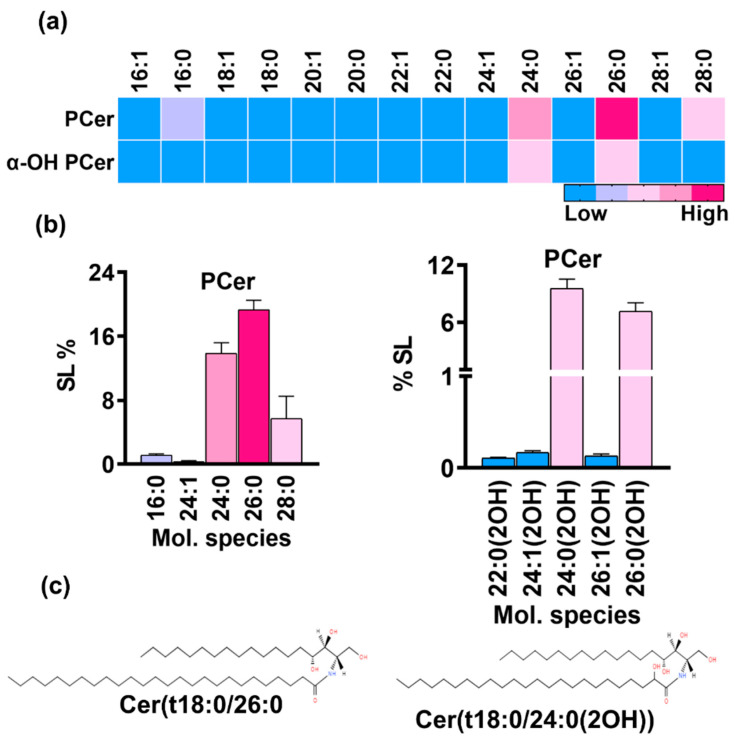
(**a**) PCer and αOH-PCer, two major classes in the acidic branch and their molecular species. (**b**) Bar graphs represents the relative amounts of top five molecular species for each class. (**c**) At the bottom, structures of major species in each class are shown.

PCer is synthesized by the acylation of PHS via the amide linkage at the C2 position of the PHS base, thus initiating the t18:0 branch of the SL biosynthesis pathway. It is the major intermediate of the acidic SL pathway. Scans of the t18:0 backbone with an *m*/*z* of 282.2 yielded masses belonging to many SL classes (Supplementary File S1). Most mass signals corresponded to species belonging to PCer. Upon quantification, PCer was revealed as the most abundant SL class with a proportion of 41% out of the total SL content. The detected PCer species prominently included an even number of carbons containing fatty acids with chain lengths of 16 to 28 carbons (Figure 6a). The significant species were 26:0, 24:0, 28:0, and 16:0, contributing 19.13%, 13.8%, 5.7%, and 1.1% to the sphingolipid content, respectively (Figure 6b). Other detected species, such as 18:0, 20:0, and 22:0, and PCer species containing mono-unsaturated fatty acids accounted for a very minute amount (<1%). Notably, the number of species with saturated fatty acids was remarkably higher than the number of ones with unsaturated fatty acids.

The hydroxylation of PCer at the C2 position of the fatty acids yields αOH-PCer, which serves as a precursor to inositol phosphorylceramide (IPC) intermediates and represents the acidic branch of SL biosynthesis in yeast and some other filamentous fungi. Acidic SLs are commonly found in several *Candida* and in other fungal species; however, IPCs were not detected in *R. delemar*. The analysis showed the accumulation of αOH-PCer, which comprised ~17% of the total SLs, thus making it the third most abundant SL class (Figure 1b). Multiple molecular species of αOH-PCer with fatty acids of varying chain lengths (14C to 28C) were detected and quantified (Figure 6a). Like PCer, major species had a similar number of carbons in fatty acids but were hydroxylated, such as 24:0(2OH) and 26:0(2OH), representing 9.5% and ~7% of the total SL content, respectively (Figure 6b). These were followed by 24:1(2OH), 26:1(2OH), and 28:0(2OH) with amounts of 0.16%, 0.13%, and 0.02%, respectively. Other species, such as 14:0(2OH), 16:0(2OH), 18:0(2OH), 20:0(2OH), and 22:0(2OH), as well as other mono-unsaturated species were only found in trace amounts (≤0.01%).

### 3.2. All Major Glycerophospholipds (GPLs) Are Present in R. delemar

**Figure 7:**
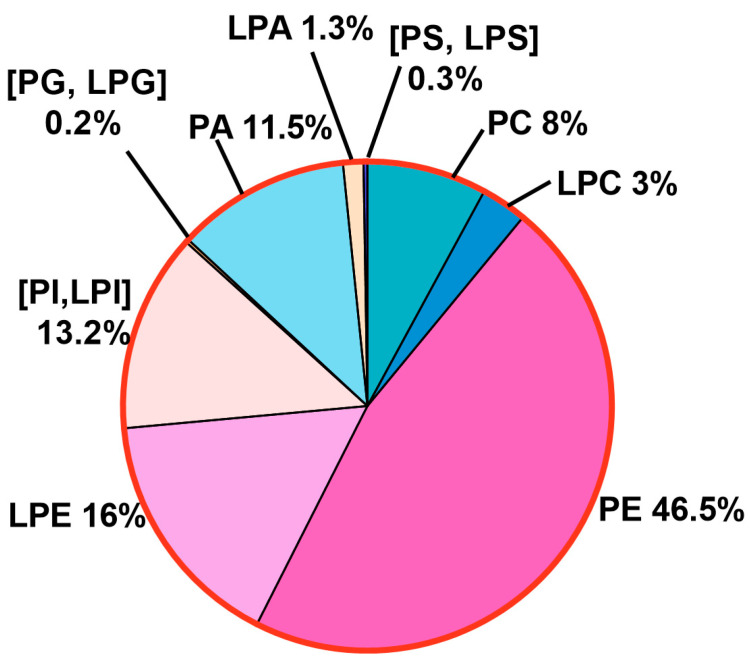
PL landscape of *R. delemar* with relative distribution of PL classes.

Considering that GPLs play diverse and important roles in the biology and pathogenesis of Mucorales, we analyzed them in great detail. Total extracted lipids were subjected to shotgun lipidomics, in which different confirmatory scans targeting the polar headgroups of GPLs were used for the detection of particular classes [36]. All scans were performed in positive mode ([M + H]^+^ or [M + NH_4_]^+^) and ranged between an *m*/*z* of 400 Da and an *m*/*z* of 1000 Da, covering all the known PL species. The different GPLs detected are given in Figure 7. Multiple species were detected that belonged to six classes of phospholipids (PLs). Approximately 200 of the top species were selected and quantified using the MRM approach, in which separate chromatograms were recorded for each species and the identification was based on the retention time with respect to the available natural standards of each class. Each class has a unique polar headgroup and one or two acyl chains linked to the glycerol backbone via ester linkages. The complete list of quantified PL species is given in Supplementary File S2.

#### 3.2.1. Phosphatidylethanolamine (PE) and Lysophosphatidylethanolamne (LPE) Constitute the Bulk of PL Content

**Figure 8:**
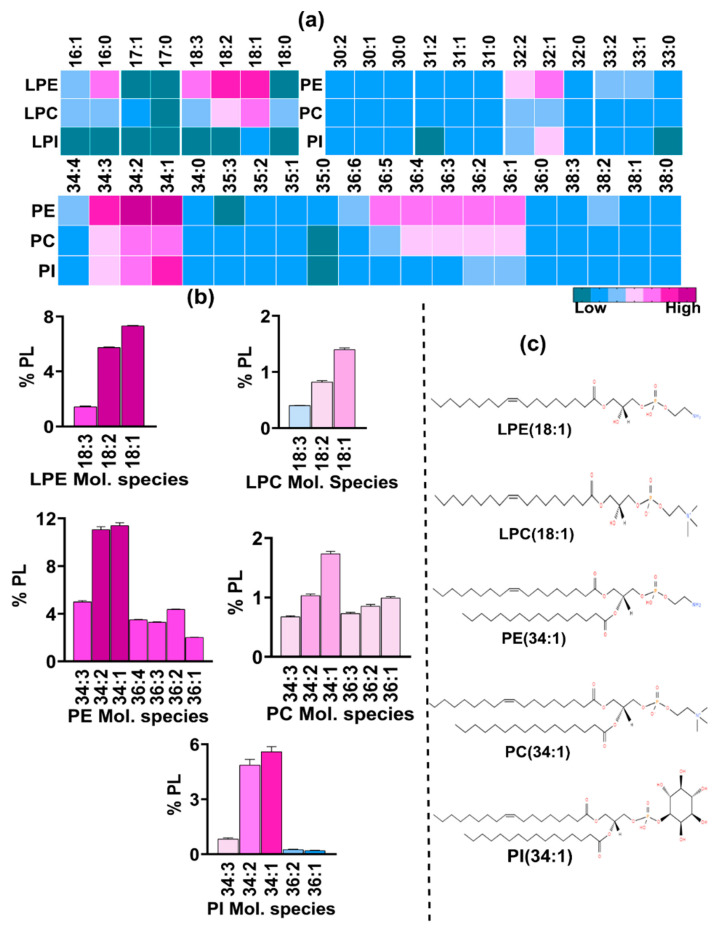
Major PL and Lyso-lipid species of different PL classes detected in *R delemar*. (**a**) Heatmap represents relative abundance of molecular species in each class with varying numbers of carbons and double bonds in both acyl chains. (**b**) Top molecular species from PC, PE, and PI were quantified and are represented by bar graphs. (**c**) Structures represent major molecular species from each class.

PL classes with the ethanolamine headgroup and two acyl chains, i.e., PE, were found to be the most abundant (46%). Together with LPE (16%) with only one acyl chain, these in combination compose the bulk of the total PL content (Figure 8). Both types of species were detected using a neutral loss scan of 141 Da. While our analysis could detect numerous PE species, those with 30 to 38 carbons and with total double bonds ranging from zero to six in both the acyl chains showed significant abundance and were quantified (Figure 8a). Notably, species with unsaturated fatty acids were more abundant than those with saturated fatty acids. For instance, the major PE species found had a total of 34C in their acyl chains, including PE 34:1 (11.6%), 34:2 (11.2%), and 34:3 (5.1%) (Figure 8b). This was followed by species with 36C, such as PE 36:2 (4.4%), 36:4 (3.4%), 36:3 (3.2%), 36:1 (~2%), 36:5 (1.8%), and 32:1 (1.8%). All other species were <1%. Species with odd-chain fatty acids with a total of 33C and 35C were also detected and were present in low amounts.

LPE species were also present in high quantities (Figure 8a). Five major species were detected, out of which the species containing 18C predominated. LPE 18:1 was the most abundant (7.3%), followed by 18:2 (5.4%) and 18:3 (1.36%) (Figure 8b). Similarly, 16C containing LPE 16:1 was the main species, followed by 16:0, comprising ~1% and 0.4% of the PL content, respectively.

#### 3.2.2. Phosphatidyl Choline (PC) Content Is Low

Unlike many fungal species, PC contents are low in *R. delemar* cells. The precursor ion scan of the phosphocholine headgroup (with an *m*/*z* of 184.1 Da) revealed the presence of species belonging to PC and LPC. Cumulatively, both types contributed ~11% to the PL content: PC contributed ~8% and LPC contributed 3%. Like PE, the species contained a total of 30C to 38C, out of which the species containing 34C, such as PC 34:1 and 34:2, were the most abundant, with proportions of 1.7% and 1%, respectively (Figure 8b). This was followed by species with 36C, such as 36:1 (~1%), 36:2 (0.85%), and 36:3 (0.73%). The other significant species included 34:3 (0.65%), 36:4 (0.56%), 32:1, and 36:5, each sharing 0.3% of the total PL content. Other species including those with saturated fatty acids and with odd-chain fatty acids of 31C, 33C, and 35C were very low (≤0.1).

Similarly, in LPC, species with saturated and unsaturated fatty acid chain lengths of 16C to 18C were detected, in which LPC 18:1, 18:2, and 18:3 were the major species, with 1.8%, 0.8%, and 0.4%, respectively, followed by LPC 16:0, 18:0, and 16:1, ranging from 0.12% to 0.16% (Figure 8b). The LPCs containing odd-chain fatty acids, 17:0 and 17:1, were found in trace amounts.

#### 3.2.3. Phosphatidyl Inositol (PI) and Phosphatidic Acid (PA) Show Equal Abundance

Lipid species with inositol as a headgroup were detected using a confirmatory neutral loss scan at 277 Da in positive mode. Multiple species belonging to PI with two acyl chains were detected and quantified (Figure 8a). However, lysophosphatidyl inositol (LPI), which has only one acyl chain, was scarce as only one LPI species showed a significant presence. All PI and LPI species constituted about 13% of the total PL content. The major species among PI were those having a total of 34 carbons in both acyl chains, such as PI 34:1, 34:2, and 34:3, contributing 5.5%, 4.8%, and 0.84%, respectively, to the total PL content (Figure 8b). These were followed by 32C, containing PI 32:1 and 32:2, with proportions of 0.5% and 0.4%, respectively. Unlike PC and PE, species containing 36C were less abundant than those with 32C. Major species in the 36C group had one to five double bonds with their levels ranging from 0.05% to 0.2%. Other species including the PI species containing odd-chain fatty acids and a single LPI species, LPI 18:1, were present in trace amounts.

**Figure 9:**
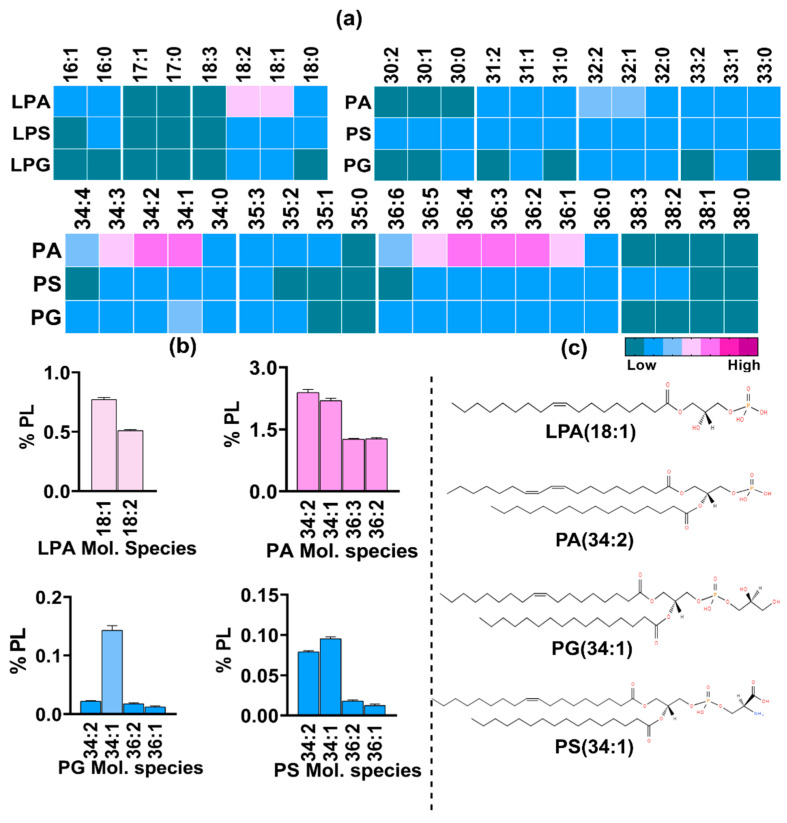
Major PL classes detected in *R delemar* and their Lyso-species. (**a**) Heatmap represents relative abundance of molecular species in each class with varying numbers of carbons and double bonds in both acyl chains. (**b**) Abundant molecular species from PA, PG, and PS were quantified and are represented by bar graphs. (**c**) Structures represent major molecular species from each class.

Similarly, lipid species with a phosphate headgroup were detected by neutral loss scanning at 115 Da and included PA and lyso-PA (Figure 9a). Their combined abundance of 12.8% was nearly equal to that of PI and LPI (~13%). The major PA species were PA 34:2, 34:1, and 34:3 with proportions of ~2.4%, 2.2%, and 0.7%, respectively (Figure 9b). These were followed by species with 36C and one to five double bonds, with their proportions ranging from 0.5% to 1.2%. Species from the 32C group, PA 32:1 and 32:2, constituted 0.45% and 0.3%, respectively. All other PA species were present in low amounts (≤0.1%).

Unlike LPI, five species of LPA had a significant presence, with the major species having 18C and 16C in their acyl chain. The major species were LPA 18:1 (0.7%), 18:2 (0.5%), 16:1 (0.04%), 16:0 (0.02%), and 18:0 (0.01%).

#### 3.2.4. Phosphatidyl Serine (PS) and Phosphatidyl Glycerol (PG) Represent the Least Abundant GPLs

In the positive mode, a neutral loss scan of 185 Da detected lipid species with the serine headgroup. Species belonging to PS with a total of 30C to 38C in their acyl chains had a significant presence (Figure 9a). The pattern was similar to that of other PL classes, with 34C-containing species being more abundant than 36C- and 32C-containing species (Figure 9b). The major species from the 34C group were PS 34:1, 34:2, and 34:3, with proportions of 0.1%, 0.08%, and 0.02%, respectively, followed by species within the 36C group, such as PS 36:2, 36:1, and 36:3 with respective amounts of 0.02%, 0.01%, and 0.01%. Among the 32C group, PS 32:1 had a significant presence (0.01%). All other PS species including saturated and odd-chain fatty acids were present in trace amounts (<0.01%). Four LPS species, LPS 16:0, 18:0, 18:1, and 18:2, were also found in minute amounts. PS and LPS in combination constituted about 0.2% of the total PL content.

Species with glycerol as a headgroup were also detected by neutral loss scanning at 189 Da and belonged to either phosphatidyl glycerol (PG) with two acyl chains or lyso PG with only one acyl chain. Similarly to PS and LPS, the total share of PG and LPG was found to be ~0.2%, hence making them the least abundant PL classes. The compositional distribution of PG species also followed the same pattern as that of other PL classes with PG 34:1 and 34:2 being the most abundant with proportions of 0.1% and 0.02%, respectively (Figure 9b). These were followed by PG 36:4 and 32:1, sharing 0.006% and 0.005%, respectively. All other species had a very low presence. Among LPG species, two species, LPG 18:1 and LPG 18:2, were quantifiable and ranged between 0.001% and 0.008%.

### 3.3. Ergosterol Is the Major Sterol and Alternative Sterol Biosynthesis Pathway Also Exists in R. delemar

**Figure 10:**
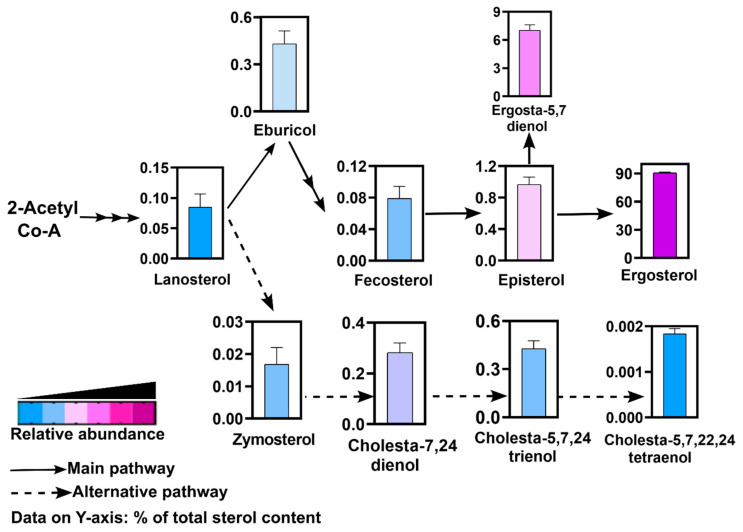
Major sterol intermediates in *R. delemar* as detected by GCMS. Only the detected intermediates are shown as bar graphs. Data on *Y*-axis represent % of each sterol intermediate of total sterol content.

We employed GCMS to detect and identify major intermediates in the sterol biosynthesis pathway in *R. delemar*. Sterols were identified as TMS derivatives, and the spectral pattern of each peak was searched against the spectral libraries of sterols [25]. While we could detect a number of TMS-derivatized sterols, some non-derivatized and unidentified sterols were also detected. Here, we selected and quantified the top sterol intermediates in which ergosterol was found to be the most abundant sterol (~90%), an observation widely recorded for the fungal kingdom (Figure 10). This was followed by ergosta-5,7 dienol (~7%) and its upstream intermediate, episterol (~1%). The first sterol in the fungal biosynthesis pathway sequence, lanosterol, was also detected. Its downstream products, eburicol and zymosterol, were also detected and were significantly abundant. Both intermediates via fecosterol lead to the synthesis of ergosterol through multiple steps.

Notably, cholesta-type sterols synthesized from zymosterol were also detected. The major cholesta-type sterols were cholesta-7,24 dienol, cholesta-5,7,24 trienol, and cholesta-5, 7, 22, 24 tetraenol. These intermediates are part of the alternative pathway of ergosterol biosynthesis in *R. delemar* (Figure 10).

## 4. Discussion

**Figure 11:**
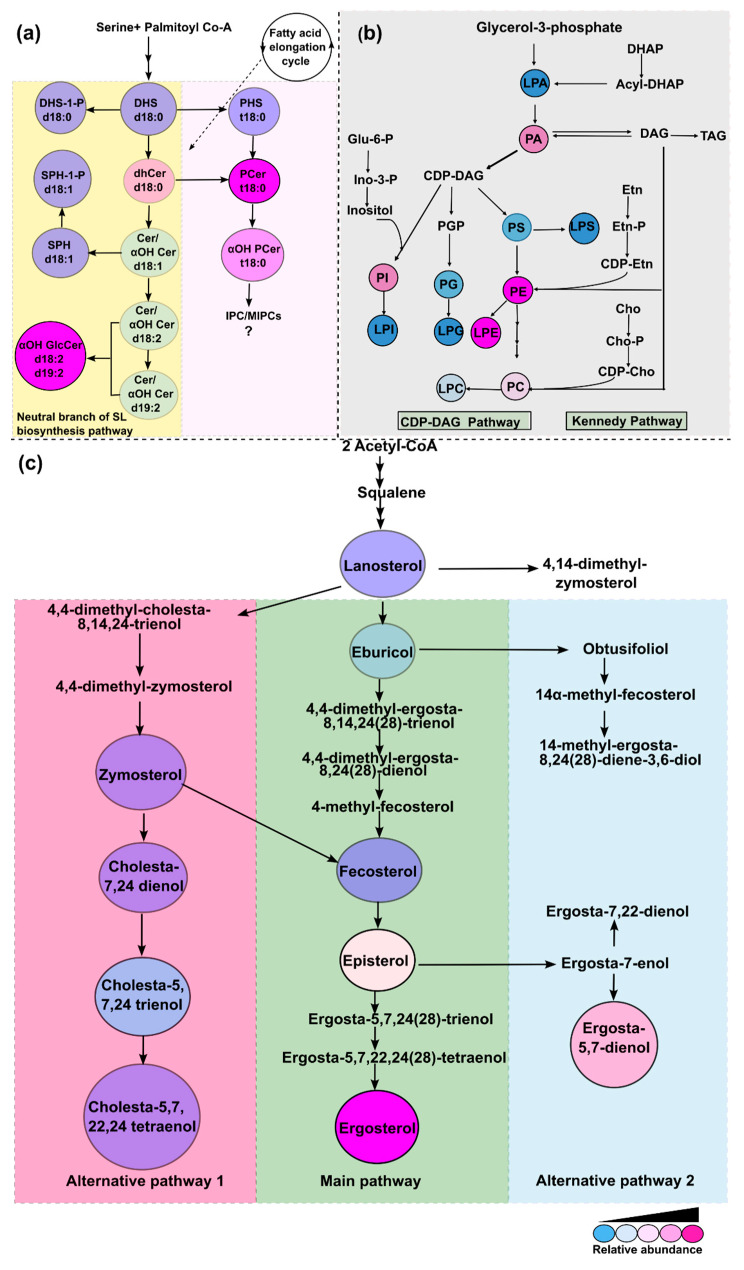
Putative lipid biosynthesis pathways in *R. delemar*. Relative abundance of each detected intermediate is depicted by different colors. (**a**) SL biosynthesis pathway based on the intermediates detected in our analyses. Description of SL class in circles is given by short abbreviations followed by characteristic backbone present. (**b**) PL biosynthesis pathway and the major classes are given in circles. (**c**) Sterol biosynthesis pathway as described in other fungi. Sterol intermediates in the circles are the abundant ones detected in our analysis. The intermediates in the central panel form the main pathway while the right and the left are alternative pathways.

Membrane lipids are targets of common antifungal agents such as azoles and polyenes. Previous studies from others and our group suggest that perturbations in the lipid profiles of fungi lead to the development of antifungal resistance [32,37,38,39,40]. Considering the intricate relationship between lipids and drug resistance, *R. delemar* 99-880, a genome sequenced isolate [21,41,42] and widely characterized representative of Mucorales, was used to study the compositional landscape of lipids. For the first time, we dissected three major lipid biosynthesis pathways, SLs, PLs, and sterols, and not only detected major intermediates of their respective pathways, but also quantified major classes and their constituent molecular species, which differed in carbon chain length and enzymatic modifications, such as hydroxylations and the degree of unsaturation. The predicted biosynthetic pathways in Figure 11 are based on the detection of the lipid intermediates and their relative abundance.

Our analysis confirms an earlier study by Aoki et al., which demonstrated evidence of AbA resistance in many Mucorales species as well as in the ascomycete fungus *Hirsutella rhossiliensis* due to the absence of typical acidic SLs, such as IPCs and mannosyl inositol phosphorylceramides (MIPCs) [28,43]. Also, neutral SL species with one or many sugar molecules in GlcCer were described in the same study [28]. However, their upstream precursors remained unexplored. In our analysis, we confirmed the presence of fungal-specific SLs of the neutral pathway with d18:2 and d19:2 sphingoid backbones that are precursors of complex GlcCers (Figure 11a).

GlcCer, as it is probably the sole complex SL in *Rhizopus,* could play a critical role. The precursors of GlcCer in fungi such as Δ8-Cer and 9-Me, Δ8-Cer are present. Both types of SLs are unique to certain fungi such as *Candida* and *Cryptococcus* and are not reported in *S. cerevisiae* and mammals [35]. In *Cryptococcus*, GlcCer has already been shown to be a major virulence factor as its depletion renders cells avirulent [44]. In other filamentous fungi, GlcCer is required for polarized growth, spore germination, and alkali tolerance, while in *C. albicans*, GlcCer mediates hyphal growth and also susceptibility to SDS and FLC [35,45,46,47]. Our recent studies have also suggested GlcCer compositional differences associated with drug resistance in clinical isolates of *C. auris* [26]. The lower abundance of upstream precursors such as d18:2 and d19:2 Cers and αOH-Cer reinforces that these intermediates are highly fluxed towards the synthesis of GlcCer to maintain its required proportion. The abundance of 16:0(2OH) in *R. delemar* differs from other fungi such as *Candida,* for which 18:0 (2OH) is the major GlcCer species [22,27]. Notably, exogenous GlcCer has been shown to antagonize the effects of a potential antifungal drug, miltefosine, in many species of Mucorales [48]. Similarly, in comparison with other yeasts such as *Candida* and *Cryptococcus*, wherein LCBs ranged between 0.5% and 1.2%, its levels are very low in *R. delemar* cells [22,26,27]. Among LCBs, DHS-1-P was found to be a major base compared to PHS and DHS, which are found to be abundant in *Candida* [26,49,50]. *LCB4*, the encoding gene for the LCB kinase, is part of Tac1 regulon, which is one of the major determinants of azole resistance in *Candida* [51]. Phosphorylated bases are known to be involved in temperature tolerance in *S. cerevisiae*, although their roles in other fungi are still unclear [52]. Also the abundance of 16:0 species in dhCer is different from other species of fungi such as *Candida* and *Cryptococcus*, in which 18:0 dhCer predominates [22,27]

The polar lipids such as PLs detected in our analysis were structurally similar to other eukaryotes. However, remarkable differences with regard to their relative composition among different PLs classes exist. For instance, the major characteristics observed in *R. delemar* PLs is a high proportion of PE as compared to lower levels of PC and a high abundance of lyso-lipids (Figure 11b). Such apparent variations reflect the completely different physiological state of *R. delemar* compared to other fungal pathogens, in which PC usually predominates over all other lipid classes [53]. Notably, a high PE content was seen to be associated with hyper-virulence in *C. albicans* [54]. In addition, a high PE:PC ratio is a major determinant of membrane rigidity that governs drug diffusion across the membrane and affects susceptibility [40,55]. Certain lyso-phospholipids were observed to be highly enriched in the drug-resistant *C. auris* isolate [49]. The high abundance of lyso-lipids such as LPE in *R. delemar* suggests strong phospholipase activity, which impacts fungal fitness and virulence. Lyso-lipids were also found in *Candida* extracellular vesicles in response to oxidative stress [56]. PLs are known to play roles in maintaining membrane rigidity, cell cycle progression, and governing stress resistance. In one of our previous studies, a drug-resistant *C. auris* isolate had higher PL levels compared to a susceptible isolate [57].

Sterol metabolism in fungi and its role in antifungal drug resistance has been a subject of research for a long time. As common antifungal drugs like azoles and polyenes target sterols, any alteration in sterol profile impacts drug susceptibility. Mutations in sterol biosynthesis genes, such as *ERG6*, *HMG1*, and *ERG3* in *Candida*, *Aspergillus*, *Mucor lusitanicus,* and *M. circinelloides,* impact the susceptibility of azoles and polyenes [31,33,58]. *Erg6* deletion leads to the synthesis of cholesta-type lipids from zymosterol via an alternative ergosterol biosynthesis pathway that is often associated with antifungal drug resistance. A genomic analysis of *R. oryzae* (*R. delemar*) [21] also reveals the presence of the genes responsible for the biosynthesis of alternative sterol intermediates, some of which were confirmed in our study as discussed earlier. Our analysis of measured sterol intermediates confirms all the reported alternative sterol pathways, which also appear to operate in *R. delemar* (Figure 11c). Also, the *R. delemar* genome contains multiple copies of several ergosterol biosynthesis genes such as *ERG11*, *ERG6*, and *ERG3*, and the gene duplication of sterol biosynthesis genes has been linked to azole resistance [21]. All these observations point towards potential resistance evolution.

In conclusion, our study presents a first draft of the landscape of all major classes of lipids in *R. delemar*, a reference species in Mucorales studies. Analyzing lipid composition among different Mucorales species, which exhibit varying levels of drug resistance, will be interesting since it could explain their diverse physiological roles and reveal their relevance in drug resistance. Nonetheless, the data provided here are expected to serve as a platform to stimulate further research on lipids, their physiological relevance, and their role in drug resistance in *Rhizopus* clinical isolates.

## Data Availability

The original contributions presented in the study are included in the article/Supplementary Material, further inquiries can be directed to the corresponding authors.

## Supplementary Materials

The following supporting information can be downloaded at: https://www.mdpi.com/article/10.3390/jof10110760/s1, Supplemental File S1: Table S1: List of confirmatory scans and lipid classes detected; Figure S1: Mass spectral signal of different confirmatory scans for detection of sphingolipids(SL) in *R. delemar* using Shot-gun lipidomics; Figure S2: Mass spectral signal of different confirmatory scans for detection of phospholipids (PL) in *R. delemar* using Shot-gun lipidomics. Supplementary File S2: Proportion of SL and PL classes and molecular species in *R. delemar* 99-880. Data represents %age of each lipid species out of total SL and PL content
