# Supplementary material for: A Comprehensive Analysis of the Lipidomic Signatures in Rhizopus delemar
Source: J Fungi (Basel). 2024 Nov 1;10(11):760. doi: 10.3390/jof10110760 (PMC11595932; doi:10.3390/jof10110760)
Supplement: Supplementary file 1 [file jof-10-00760-s001.zip › Supplemental File S1.pdf]

Table S1: List of confirmatory scans and lipid classes detected.

	**Scan type**	**Backbone/ headgroup**	**Lipid Class/ Sub-class**
SL	Prec +262.2	d18:2	Δ8-Cer/Δ8- αOH-Cer
	Prec +264.1	d18:1	Cer, αOH-Cer
	Prec +266	d18:0	DHS, dhCer
	Prec +276.2	d19:2	9-Me Δ8-Cer, 9-Me Δ8- αOH-Cer, αOH-GlcCer
	Prec +282.2	t18:0	PHS, PCer, αOH-PCer
PL	Prec +184.1	PC*	PC, LPC
	NL 141	PE*	PE, LPE
	NL 277	PI*	PI, LPI
	NL 115	PA*	PA, LPA
	NL 185	PS*	PS, LPS
	NL 189	PG*	PG, LPG

* head group scan

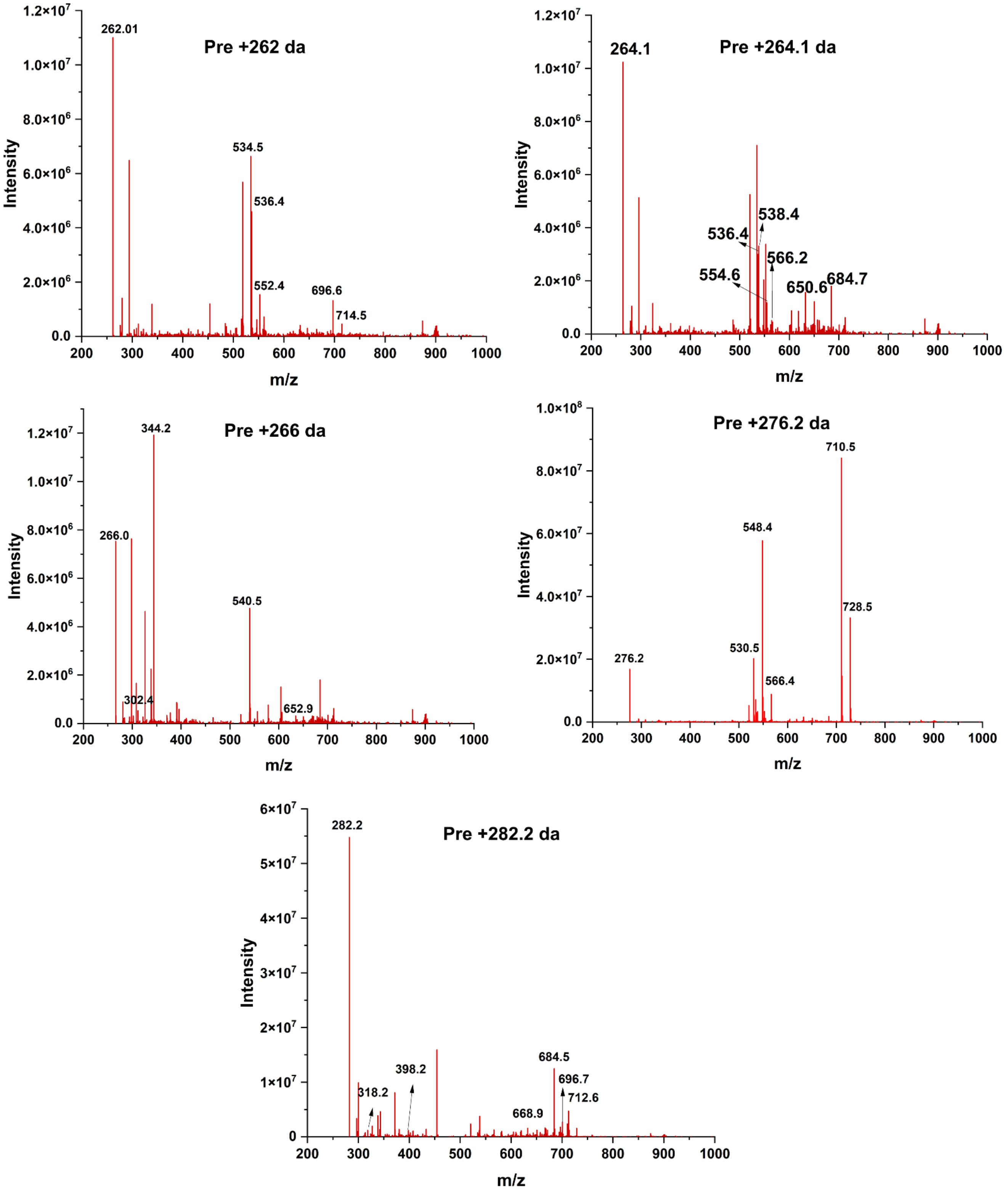

Fig S1: Mass spectral signal of different confirmatory scans for detection of sphingolipids(SL) in *R. delemar* using Shot-gun lipidomics*.* Each scan was performed over a range of 200 Dalton to 1000 Dalton with different acquisition parameters. Precursor Ion scan of m/z 262, 264.1, 266,276.2 and 282.2 da was performed for detection of SLs with d18:2, d18:1, d18:0, d19:2 and t18:0 respectively. All scans were performed in positive ($M+H^+$) mode

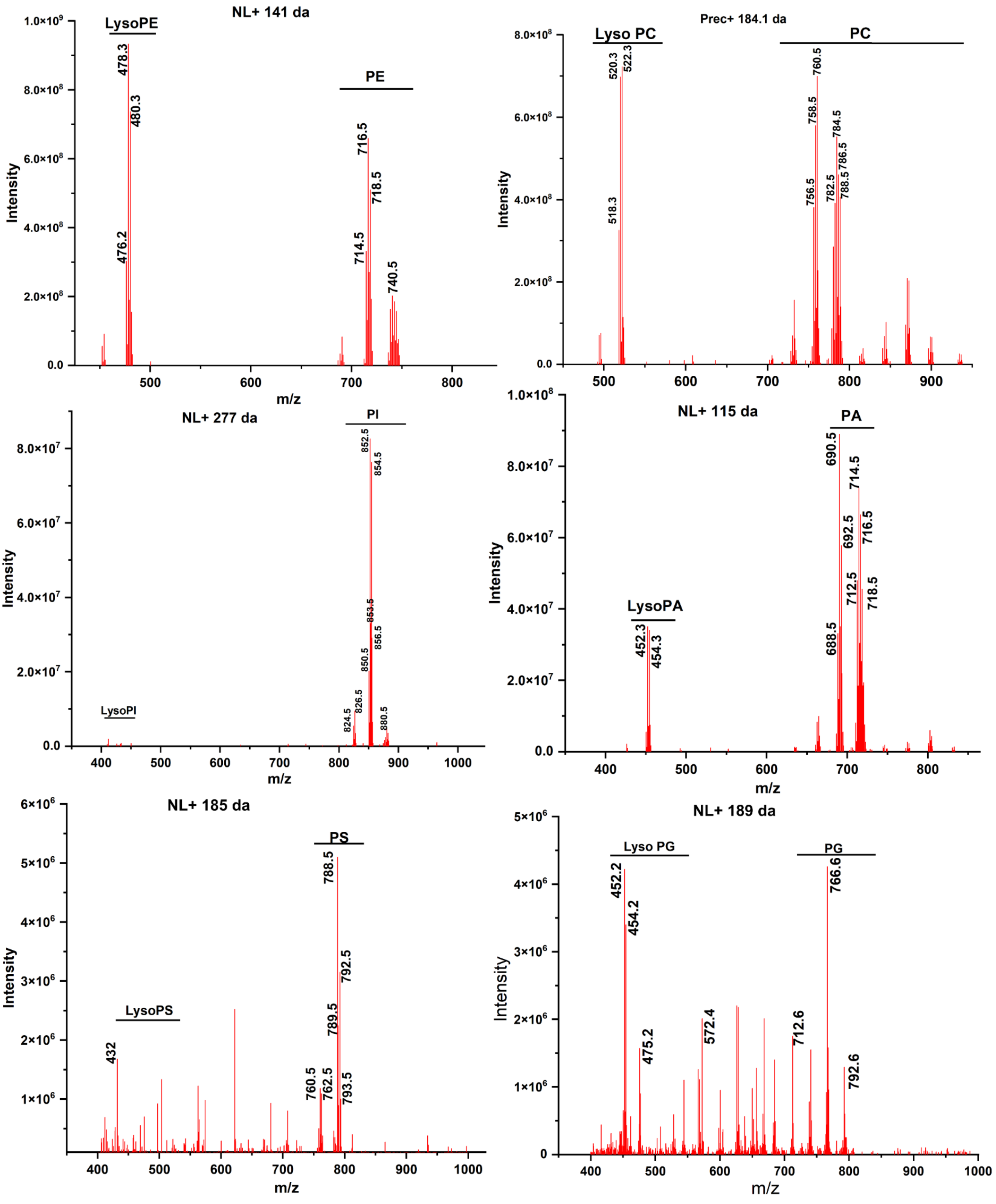

Fig S2: Mass spectral signal of different confirmatory scans for detection of phospholipids(PL) in *R. delemar* using Shot-gun lipidomics. Each scan was performed over a range of 400 dalton to 1000 dalton with different acquisition parameters. Precursor Ion (Prec) scan of m/z +184.1 was used for detection of PC while as PE, PI, PA, PS and PG were detected by using Neutral Loss(NL) scans of 141, 277, 115, 185 and 189 dalton respectively. All scans were performed in positive ($M+H^+$ or $M+NH_4^+$) mode
